# Vaccine Candidate Double Mutant Variants of Enterotoxigenic *Escherichia coli* Heat-Stable Toxin

**DOI:** 10.3390/vaccines10020241

**Published:** 2022-02-04

**Authors:** Ephrem Debebe Zegeye, Yuleima Diaz, Pål Puntervoll

**Affiliations:** 1 NORCE Norwegian Research Centre, Postboks 22 Nygårdstangen, 5838 Bergen, Norway; yudi@norceresearch.no (Y.D.); papu@norceresearch.no (P.P.); 2 Centre for International Health, Department of Global Public Health and Primary Care, University of Bergen, Postboks 7804, 5020 Bergen, Norway

**Keywords:** enterotoxigenic *Escherichia coli* (ETEC), heat-stable enterotoxin (ST), diarrhea, toxoid, vaccine, cross-reaction, nanovaccine, dmLT, double mutant toxoid, neoepitope

## Abstract

Heat-stable enterotoxin (ST) producing enterotoxigenic *Escherichia coli* (ETEC) strains are among the top four enteropathogens associated with moderate-to-severe diarrhea in children under five years in low-to-middle income countries, thus making ST a target for an ETEC vaccine. However, ST must be mutated to abolish its enterotoxicity and to prevent a potential immunological cross-reaction due to its structural resemblance to the human peptides uroguanylin and guanylin. To reduce the risk of eliciting cross-reacting antibodies with our lead STh-A14T toxoid, L9 was chosen as an additional mutational target. A double mutant vaccine candidate immunogen, STh-L9A/A14T, was constructed by conjugation to the synthetic virus-like mi3 nanoparticle using the SpyTag/SpyCatcher technology. This immunogen elicited STh neutralizing antibodies in mice, but with less consistency than STh-A14T peptide control immunogens. Moreover, individual sera from mice immunized with both single and double mutant variants displayed varying levels of unwanted cross-reacting antibodies. The lowest levels of cross-reacting antibodies were observed with STh-L9K/A14T control immunogens, suggesting that it is indeed possible to reduce the risk of eliciting cross-reacting antibodies by mutation. However, mutant-specific antibodies were observed for most double mutant immunogens, demonstrating the delicate balancing act between disrupting cross-reacting epitopes, keeping protective ones, and avoiding the formation of neoepitopes.

## 1. Introduction

Enterotoxigenic *Escherichia coli* (ETEC) infection accounts for over 50,000 human deaths annually [1]. ETEC represents a genetically diverse group of *E. coli* strains defined by the secretion of heat-stable enterotoxin (ST) and/or heat-labile enterotoxin (LT). Importantly, ST-producing ETEC (ST-ETEC) strains with or without LT are among the top four enteropathogens associated with moderate-to-severe diarrhea (MSD) [2] as well as the more common less severe diarrhea [3] in children under the age of five in low- and middle-income countries. Moreover, ST-ETEC infection increases the risk of death in children younger than 24 months with MSD [4], as well as contributing to long-term sequelae associated with diarrhea in these children [5,6]. In addition, there is evidence that ST may reduce the ability to mount an effective immune response to other infectious agents [7]. ETEC colonize the small intestine via adhesins, also known as colonization factors (CFs) [8]. Colonization allows ST-ETEC to effectively deliver ST to the intestinal guanylyl cyclase C (GC-C) receptor, prompting a signaling cascade that leads to the disruption of water and electrolyte homeostasis, which may ultimately lead to a profuse watery diarrhea. ST activates the GC-C receptor with higher potency than the endogenous ligands, uroguanylin and guanylin, which regulate water absorption and the hydration of the intestines [9,10]. ETEC strains infecting humans can carry either or both of two subtypes of ST, namely the 19-amino acid human ST (STh) and the 18-amino acid porcine ST (STp) [11,12]. STh and STp share 14 amino acids and have highly similar structures due to three shared structure-defining disulfide bonds [11,13].

To curb the disease burden of ETEC, efforts are underway to develop broadly protective vaccines [14], but to date, there is no licensed ETEC vaccine. Two major vaccine candidates that aim to induce immune responses to LT and the major CFs are the live-attenuated ACE527 [15] and the inactivated whole cell ETVAX [16]. However, choosing which CFs to include in a vaccine to obtain the broadest possible coverage is complicated by the fact that ETEC strains may produce one or more of over 25 distinct CFs [8,17], and that new CFs are likely to be discovered. The important observations that ST-producing ETEC are more closely associated with childhood diarrhea [2,12] and risk of death in MSD children younger than 2 years [4] strongly suggest that the highly conserved STs should be targeted in an ETEC vaccine that aims to offer broad protection. 

No natural immunity to the ST toxins have been observed, most likely due to their small sizes (~2 kDa), which implies that they must be made immunogenic. Several strategies for making ST immunogenic have been pursued, including coupling it to protein carriers by genetic fusion or chemical conjugation [11]. The advantage of the latter approach is that the ST peptides can be subjected to thorough biochemical and biophysical characterizations to ensure that protective epitopes are intact prior to conjugation. Another advantage is that higher hapten-to-carrier ratios can be obtained with conjugation than with genetic fusions. We recently conjugated STh to the coat protein of Acinetobacter phage AP205 by using the SpyCatcher system [18], resulting in virus-like particles (VLPs) carrying up to 180 copies of STh per VLP [19]. These STh-carrying VLPs were highly immunogenic in mice, and resulting serum antibodies completely neutralized the toxic activities of native STh. An attractive alternative to VLPs are the SpyCatcher-mi3 nanoparticles, which are based on a computationally designed porous dodecahedral i301 60-mer [20], which was rationally engineered for improved particle uniformity, stability, and yield [20]. Indeed, the SpyCatcher-mi3 nanoparticles were recently shown to be a promising platform for delivering SARS-CoV-2 candidate antigens [21].

To generate a safe vaccine component from ST, it must be made non-toxic. Although coupling ST to a carrier will reduce its toxicity, mutation is required to make it completely non-toxic [11]. However, due to the small size of the ST peptide, any mutation risks disrupting protective epitopes required to elicit neutralizing antibodies. Despite this, important progress has been made on identifying ST mutant variants with reduced or abolished toxicity that can elicit neutralizing antibodies (ST toxoids) when coupled to a carrier [22,23,24]. In a screen of all possible 361 single-amino acid mutations of STh to identify variants with no or low toxicity and intact epitopes, the top 30 candidates all had mutations of residues A14, N12, and L9 [22]. The STh-A14T mutant variant was shown to reduce toxicity more than 800-fold without disrupting an Y19-dominated neutralizing epitope [25], and when coupled to AP205 VLPs, the STh-A14T mutation did not seem to negatively impact the ability to elicit ST neutralizing antibodies [19]. 

A final and equally important challenge in the pursuit of a safe ST vaccine component is to avoid eliciting antibodies that cross-react with the endogenous GC-C ligands, uroguanylin and guanylin [26,27]. Both native STh and STp can engender such antibodies when made immunogenic by chemical conjugation to bovine serum albumin (BSA) [27]. Epitope mapping of 13 neutralizing anti-STh or anti-STp monoclonal antibodies (mAbs) have shed light on the nature of cross-reacting epitopes [22,27]. Although most epitopes appeared to have at least one amino acid residue shared with guanylin or uroguanylin, only two mAbs displayed demonstrable cross-reactivity to the endogenous peptides [22,27]. The major epitope residue for both of these cross-reacting mAbs was L9, which is shared with uroguanylin, and L9 was also a prominent epitope residue for an anti-STh rabbit serum that partially cross-reacted with uroguanylin [25,26,27]. This implies that L9 is a prime mutational target for reducing the risk of eliciting unwanted cross-reactivity. Interestingly, very low levels of cross-reacting antibodies were observed in sera from mice immunized with AP205 VLPs carrying native STh or STh-A14T [19]. This suggests that also choice of carrier and antigen presentation may impact the risk of eliciting cross-reacting antibodies.

The aim of this study was to construct an effective STh-based vaccine candidate with a reduced risk of eliciting antibodies that cross-react with uroguanylin and guanylin. To this end, we conjugated STh-A14T and STh-L9A/A14T with N-terminal SpyTags to SpyCatcher-mi3 nanoparticles. As controls, we constructed immunogens from untagged STh-A14T, STh-L9A/A14T, STh-L9N/A14T, and STh-L9K/A14T by chemical conjugation to the frequently used BSA carrier. The immunogens were used to immunize mice, and the resulting sera were characterized for anti-STh and carrier-specific antibody titers, their ability to neutralize STh, and immunological cross-reaction to STp, guanylin, and uroguanylin. 

## 2. Materials and Methods

### 2.1. Construction, Expression, and Purification of STh Mutant Peptides 

The pET-DsbC-STh-A14T [25] and pET-DsbC-SpyT-A14T [19] plasmids were used to express untagged and SpyTag (SpyT) tagged STh-A14T peptides, respectively. Plasmids for the expression of untagged STh-L9A/A14T, STh-L9N/A14T, and STh-L9K/A14T, and tagged SpyT-STh-L9A/A14T were made using the Q5^®^ site-directed mutagenesis Kit (New England Biolabs, Ipswich, UK), the primers listed in Table 1, and the parent plasmids listed in Table 2. All resulting plasmids were verified by sequencing and are listed in Table 2. Untagged STh mutant peptides were expressed and purified as previously described [25]. Briefly, plasmids were transformed into *E. coli* BL21 Star™ (DE3) (Invitrogen, Waltham, MA, USA), cultivated in 2YT medium supplemented with 2% (*w*/*v*) glucose and 50 µg/mL kanamycin, and induced using 0.5 mM IPTG. After expression, cells were lysed using lysozyme and ultrasonication, and after removal of cell debris by centrifugation, the cleared lysates were subjected to Ni-NTA purification. Next, the STh mutant peptides were cleaved off from their DsbC fusion partner using the Tobacco Etch Virus (TEV) protease. The fusion partner was removed using a second round of Ni-NTA purification, and the peptide-containing flow through was subjected to reversed-phase chromatography. Fractions corresponding to distinct peaks were pooled, and a rotary evaporator (Rotavapor^®^ R-100, Buchi, Flawil, Switzerland) was used to remove methanol and concentrate the samples. The masses of the purified STh mutant peptides were confirmed using matrix-assisted laser desorption/ionization time-of-flight (MALDI-TOF) mass spectrometry (MS), and the isomer with correct disulfide bridge connectivity was identified using competitive ELISA (both methods are described below). The SpyTag-tagged STh mutant peptides were also expressed and purified as previously described [19]. The purification protocol is identical to the one described above, except that size-exclusion chromatography (HiLoad^®^ 16/600 Superdex^®^ 30 pg, GE Healthcare Bio-Sciences, Uppsala, Sweden) replaced the reversed-phase chromatography. Distinct peaks were pooled and concentrated using 3 kDa cut-off Amicon Ultra-15 centrifuge filters. The correct peak was identified using competitive ELISA and MALDI-TOF MS.

### 2.2. Mass Spectrometry 

MALDI-TOF MS analysis was performed by the Proteomics Service facility at the University of Oslo, Norway, as described previously [25]. Briefly, the peptides were analyzed using an ULTRAFLEX II (Bruker Daltonics, Bremen, Germany) MALDI-TOF/TOF mass spectrometer in positive ion reflector mode. 0.4 µL sample was mixed with 0.4 µL alpha-cyano-4-hydroxycinnamic acid (Bruker Daltonics) matrix solution (10 mg/mL in 0.1% trifluoroacetic acid/acetonitrile) in a 1:1 ratio and spotted onto a MALDI plate. The mass spectra were internally calculated from the raw mass spectra using the SNAP algorithm in FlexAnalysis 2.4 (Bruker Daltonics) and compared to theoretical masses.

### 2.3. STh Mutant Peptide Antigenicity by Competitive ELISA 

Competitive ELISAs were performed essentially as described previously [26]. Nunc Immobilizer Amino Plates (Thermo Fisher Scientific, Waltham, MA, USA) were coated with 4 ng native STh per well in 100 µL PBS buffer (3.25 mM Na_2_HPO_4_, 9.6 mM NaH_2_PO_4_, 146 mM NaCl [pH 7.4]) overnight at 4 °C. All subsequent incubations were performed at room temperature. The wells were emptied and blocked with 180 µL 1% ovalbumin (A5503, Sigma, St. Louis, MO, USA) in PBS-T buffer (PBS, 0.05% Tween-20) for 1 h, followed by 3 washes with PBS-T. Next, 60 µL three-fold serially diluted competing peptide (concentrations ranging from 10 µM to 0.17 nM) were added to wells in triplicate, together with 60 µL anti-ST antibody. Competing peptides used in this study were STh (positive control), STh-A14T, STh-L9A/A14T, STh-L9N/A14T, STh-L9K/A14T, SpyT-STh-A14T, and SpyT-STh-L9A/A14T. Antibodies used were the anti-STp C30 (clone M120530 [Fitzgerald, North Acton, MA, UK]) and anti-STh 7E52 [27] monoclonal antibodies at 1:16,000 and 1:30 dilutions, respectively. Blank (B) wells were incubated with 120 µL PBS-T and total activity (TA) wells were incubated with 60 µL anti-ST antibody and 60 µL PBS-T. Competition proceeded for 1 h 45 min with gentle shaking after which the wells were emptied and washed 3 times with PBS-T. Next, 100 µL 1:4000-diluted alkaline phosphatase-conjugated rabbit anti-mouse IgG secondary antibody (Abcam, Cambridge, UK) was added. After 1 h incubation, the wells were emptied, washed 3 times with PBS-T, and incubated with 100 µL of substrate (0.5 mg 4-Nitrophenyl phosphate disodium salt hexahydrate [Sigma-Aldrich Co., St. Louis, MO, USA] per ml of diethanolamine buffer pH 9.8 [Sigma]). After approximately 20 min, absorbance at 405 nm was measured using a microplate reader (Hidex, Turku, Finland). The percent inhibition of maximum binding was calculated from the absorbances of the sample well (A), the average of the blank wells (B) and the average of the total activity wells (TA) using the formula (1−(A−B)/(TA−B)) × 100. The 50% inhibitory concentration (IC_50_) for a given peptide can be used as a measure of its antigenicity. We calculated the IC_50_ values using four-parameter logistic regression analyses in Prism 8 (GraphPad Software, La Jolla, CA, USA). No constraints were used for the untagged peptides, but for the tagged peptides, the bottom parameter was set to zero and the top parameter was set to 100. Ordinary one-way ANOVA and Tukey’s multiple comparison test was used to test whether the IC_50_ for a given mutant peptide was significantly different from that of native STh. 

### 2.4. STh Mutant Peptide Toxicity by T84 Cell Assay 

The T84 cell assay was performed essentially as described previously [26]. Briefly, T84 cells (ATCC, Rockville, MD, USA) were seeded and grown to confluence on 48-well plates (Nunc, Roskilde, Denmark) in Dulbecco’s Modified Eagle Medium/Nutrient Mixture F-12 (DMEM-F12) (Gibco life technologies, Paisley, UK), supplemented with 10% fetal bovine serum (Sigma-Aldrich) and 0.2% gentamicin (LONZA, Walkersville, MD, USA). The cells were washed 3 times with 250 µL DMEM-F12 and pre-incubated with 40 µL DMEM-F12 containing 1 mM 3-isobutyl-1-methylxanthine (Sigma-Aldrich) for 10 min at 37 °C. A volume of 40 µL 1 µM native or mutant STh peptide was added to each well in duplicate (final peptide concentration 0.5 µM) and incubated for 30 min at 37 °C. Following incubation, the reaction medium was aspirated, and the cells were lysed with 0.1 M HCl at 20 °C for 20 min. Subsequently, the lysates were centrifuged at 16,000× *g* for 10 min, and the supernatants were collected to estimate cGMP levels using a cGMP enzyme immunoassay kit (Enzo Life Sciences, Inc., Farmingdale, NY, USA). The analysis was conducted according to the manufacturer’s instructions.

### 2.5. Construction of STh Mutant Peptide-mi3 Nanoparticle Immunogens

Conjugation of SpyTag-tagged STh mutants to mi3 nanoparticles was performed by mixing 1 mg SpyCatcher-mi3 (mi3-SpyC) [20], a kind gift from Mark Howarth (University of Oxford, Oxford, UK), with 3 molar excess of SpyT-STh-A14T or SpyT-STh-L9A/A14T in conjugation buffer (1.8 mM KH_2_PO_4_, 10 mM Na_2_HPO_4_, 2.7 mM KCl, 137 mM NaCl, pH 7.4) in ~2 mL final volume. The conjugation reactions were carried out for 16 h at room temperature with gentle shaking. As a negative control, mi3-SpyC in PBS buffer was incubated under the same condition. After the conjugation reaction reached completion (progress monitored by running aliquots of the reaction mixture on SDS-PAGE), excess peptides and mi3-SpyC monomers (if any) were removed by dialyzing against 2 l conjugation buffer supplemented with Tween-20 (conjugation buffer, 0.1% Tween-20) using a 300 kDa MWCO dialysis membrane (Spectrum Laboratories, Inc., Rancho Dominguez, Los Angeles, CA, USA) for 4 h initially, and later with a fresh buffer for an additional ~15 h at 4 °C. Next, endotoxins were removed by Triton X-114-based phase separation as described previously [29]. Briefly, Triton X-114 (Sigma-Aldrich) was added to aliquots (~ 600 µL) of the conjugates in endotoxin-free microcentrifuge tubes to a final concentration of 1% and allowed to dissolve on ice. Next, the mixtures were incubated on ice for 5 min at 37 °C and centrifuged at 16,900× *g* for 1 min. The supernatant was carefully removed, and the entire procedure was repeated twice. Endotoxin concentrations were measured using the Pierce™ LAL Chromogenic endotoxin quantitation kit (Thermo Scientific, Rockford, IL, USA). Endotoxin levels were estimated be 0.05 and 0.03 EU/mL for the mi3-SpyC:SpyT-STh-A14T and mi3-SpyC:SpyT-STh-L9A/A14T nanoparticles, respectively. The final conjugate concentrations were measured using the Pierce™ BCA Protein Assay Kit (Thermo Scientific) according to the manufacturer’s instructions.

### 2.6. Construction of STh Mutant Peptide BSA Conjugate Immunogens 

Chemical conjugation of untagged STh mutant peptides to BSA was performed by GenScript (Leiden, The Netherlands). BSA (Amresco 0332, Quality Biological Inc., Gaithersburg, MD, USA) was dissolved in PBS to a concentration of 10 mg/mL and mixed with selected STh mutant peptides (STh-A14T, STh-L9A/A14T, STh-L9K/A14T or STh-L9N/A14T) at 1:1 mass ratios, followed by dilution with PBS to half the final reaction volume (1 mg/mL). Next, 0.25% glutaraldehyde (Sinopharm Chemical Reagent Co., Ltd. cat # 30092436, Shanghai, China) (half final volume) was slowly added and placed in a constant temperature oscillator at 25 ± 2 °C for 2 h. To quench unreacted glutaraldehyde, 1% glycine was subsequently added and further incubated at 25 ± 2 °C for 1 h in dark. To remove STh mutant peptides not covalently linked to BSA, the conjugate samples were dialyzed against PBS using a 14 kDa MWCO dialysis membrane at room temperature for 2 h, followed by a change of fresh PBS buffer and overnight (~15 h) at 4 °C.

### 2.7. SDS-Polyacrylamide Gel Electrophoresis (SDS-PAGE) and Immunoblots 

SDS-PAGE was performed using 4–20% gradient mini-PROTEAN TGX™ gels (BioRad Laboratories Inc., Hercules, CA, USA) run at 200 V for 35 min. Subsequently, proteins were either stained using SimplyBlue™ safe Stain (Novex, Life Technologies, Carlsbad, CA, USA) or transferred onto 0.45 µm nitrocellulose membrane (BioRad) for immunoblots. Immunoblotting was performed with a tank blotting system (4 °C, 100 V, 1 h, gentle stirring) and the transfer buffer 25 mM Tris, 192 mM glycine, 20% ethanol, pH 8.3 (BioRad). Next, the membrane was washed 3 times for 5 min with TBS-T buffer (20 mM Tris, 150 mM NaCl, 0.1% Tween-20, pH 7.5) after each of the following steps: the membrane was blocked with 3% skim milk powder (Fluka Analytical, Darmstadt, Germany) in TBS-T for 1 h, incubated with 1:3000 C30 monoclonal antibody (10–1014, Fitzgerald Industries International, North Acton, MA, USA) overnight at 4 °C, and incubated with 1:3000 anti-mouse ECL antibody (NA931, GE Healthcare Life Sciences) for 1 h. Finally, the immunoblot was developed using ECL substrate (BioRad) and imaged using ChemiDoc ™ XRS+ imaging system (BioRad).

### 2.8. Transmission Electron Microscopy (TEM) of mi3-SpyC:SpyT-STh-L9A/A14T Nanoparticles 

TEM was performed by the Molecular Imaging Centre Core Facility of the University of Bergen, Norway. Briefly, 4 µL mi3-SpyC:SpyT-STh-L9A/A14T nanoparticles (10 µg/mL) were pipetted onto a carbon 200 mesh copper grid for 2 min, blotted with filter paper, and air dried. Next, the samples were negatively stained twice with a freshly prepared 2% uranyl acetate for 10 s, blotted, and air-dried. Finally, the samples were visualized using TEM (Jeol JEM-1230) running at 80 kV and imaged with a Gatan multiscan camera (model 791).

### 2.9. Estimation of Hapten-Carrier Ratio by Amino Acid Analysis 

Amino acid analysis of the BSA-STh mutant conjugates was performed by the Amino Acid Core Facility at Chemistry of Biomolecules Unit, Institute Pasteur, Paris, France. The conjugates (40 µL) were hydrolyzed using 6N HCl supplemented with 1% phenol in glass tubes for 48 h at 110 °C in the presence of a known amount of the internal standard norleucine. After evaporation of the HCl, the samples were analyzed for amino acid composition using an amino acid analyzer (Hitachi L-8800) [30]. Calculations were performed using Phe as reference, as there are 27 Phe in BSA, and none in the peptides. The peptide content calculation was done based on the most reliable amino acids (Asp, Glu, Gly, Ala, and Leu). Hapten-carrier ratios are reported as the average (±standard deviation) estimation based on calculations for each of at least three amino acids.

### 2.10. Mouse Immunizations 

Mouse immunizations were conducted using 9-week-old female BALB/c mice by GenScript (The Netherlands) in AAALAC international/OLAW accredited labs. 

The mi3 nanoparticles were used to immunize groups of five mice subcutaneously. Prior to injections, prime doses were prepared by mixing 1.25 nmol mi3-SpyC:SpyT-STh-A14T or mi3-SpyC:SpyT-STh-L9A/A14T, corresponding to ~2.5 µg mutant STh peptide, with 1 µg of the double mutant heat-labile toxin (dmLT) adjuvant [31]. An additional group of mice was immunized with 1.25 nmol mi3-SpyC:SpyT-STh-L9A/A14T without adjuvant. Two booster doses were administered in two-week intervals following the prime dose, and the final blood specimen was collected from the heart two weeks after the administration of the final booster dose (day 42). 

The following BSA-conjugates were used to immunize groups of five mice intraperitoneally: BSA:STh-A14T, BSA:STh-L9A/A14T, BSA:STh-L9N/A14T or BSA:STh-L9K/A14T. Prior to injections, prime doses were prepared by mixing 50 µg of each conjugate with Freund’s complete adjuvant (1:1 *v*/*v*), and booster doses were prepared by mixing 25 µg conjugates with Freund’s incomplete adjuvant (1:1 *v*/*v*). Three booster doses were administered in two-week intervals following the prime dose, and the final blood specimen was collected from the heart two weeks after the administration of the final booster dose (day 56). 

### 2.11. Estimation of Serum Antibody Titers 

Endpoint serum antibody titers were measured by ELISAs as described previously [19]. Briefly, Nunc Immobilizer Amino Plates (Thermo Fisher Scientific, Waltham, MA, USA) were coated with 40 ng STh (native or mutant STh peptide as indicated), BSA, mi3-SpyC, or dmLT per well in 100 µL PBS overnight at 4 °C for anti-STh, anti-BSA, anti-mi3-SpyC, and anti-dmLT titer estimation, respectively. All subsequent incubations were performed at room temperature. The wells were emptied and blocked with 180 µL 1% ovalbumin (A5503, Sigma) in PBS-T for 1 h, followed by 3 washes with PBS-T. Next, 120 µL two-fold serial dilutions of each mouse serum (1:1000–1:2,048,000) were applied to the wells in triplicate and incubated at room temperature for 1 h. PBS-T only was added to blank wells as background signal controls. After washing 3 times with PBS-T, 100 µL 1:4000 alkaline phosphatase-labelled rabbit anti-mouse IgG (ab6729, Abcam) was added to each well and incubated for 1 h, followed by another 3 washes with PBS-T. Next, substrate (0.5 mg 4-Nitrophenyl phosphate disodium salt hexahydrate [Sigma] per ml of diethanolamine buffer pH 9.8 [Sigma]) was added to each well, and after 30 min absorbance at 405 nm was measured using a microplate reader (Hidex, Finland). The antibody titers were defined as the highest dilution in each series with a signal-to-background ratio ≥ 2.1. Prism 8 (GraphPad Software, La Jolla, CA, USA) was used to perform ordinary one-way analysis of variance (ANOVA) and Tukey’s multiple comparison test to evaluate whether serum antibody titer means were significantly different. 

### 2.12. Neutralization of Native STh in T84 Cell Assay 

To test the ability of the individual mouse sera to neutralize the toxicity of STh, the sera were diluted 1:10 in DMEM/F-12 medium, mixed with native STh to a final concentration of 10 nM, and incubated for 2 h at 37 °C. Next, the samples were tested in the T84 cell assay as described above. The serum with an anti-STh titer lower than 250 and the highest apparent neutralizing ability was chosen as a control in a two-way ANOVA, followed by Fishers Least Significant Difference (LSD) test, to determine true neutralizing activity, i.e., only sera significantly different (*p* < 0.05) from the control were considered to be neutralizing. The statistical analysis was performed using Prism 8 (GraphPad Software, La Jolla, CA, USA).

### 2.13. Immunological Cross-Reactivity by Competitive ELISA 

To assess the immunological cross-reactivity of the mouse sera, we tested the ability of STp, uroguanylin, and guanylin, compared to that of STh, to outcompete binding of those sera to immobilized STh in a competitive ELISA performed as described above. Cross-reacting fractions were calculated for STp, uroguanylin, and guanylin as previously described [19,27]. Briefly, four-parameter log-logistic regression models were generated using the drc package in R [32] with the bottom parameter set to 0 and the top parameter set to a maximum value of 100. The fitted models were used to calculate the 90 percent inhibitory concentrations (IC_90_) for STh, and those concentrations were used to calculate the percent inhibition of binding for each of the other peptides. The estimated values were adjusted by subtracting the bottom parameter estimate, and IC_90_ cross-reacting fractions were calculated by dividing the adjusted percent inhibition value of each peptide by the corresponding inhibition values for STh. Sera with anti-STh titers < 4000 could not be tested for cross-reactivity. 

## 3. Results and Discussion

### 3.1. Purification and Characterization of Double Mutant STh Peptides 

Informed by our screen of all possible single-amino-acid mutants of STh [22], we chose STh-L9A/A14T as the prime double mutant STh (dmSTh) candidate. To facilitate conjugation to SpyCatcher-mi3 (mi3-SpyC) nanoparticles, we constructed variants of the STh-L9A/A14T and STh-A14T peptides with N-terminal SpyTags [19]. In addition, untagged constructs of the same peptides, as well as STh-L9N/A14T and STh-L9K/A14T, were made. All peptides were recombinantly expressed and purified, resulting in preparations with correct molecular masses as confirmed by mass spectrometry.

**Figure 1:**
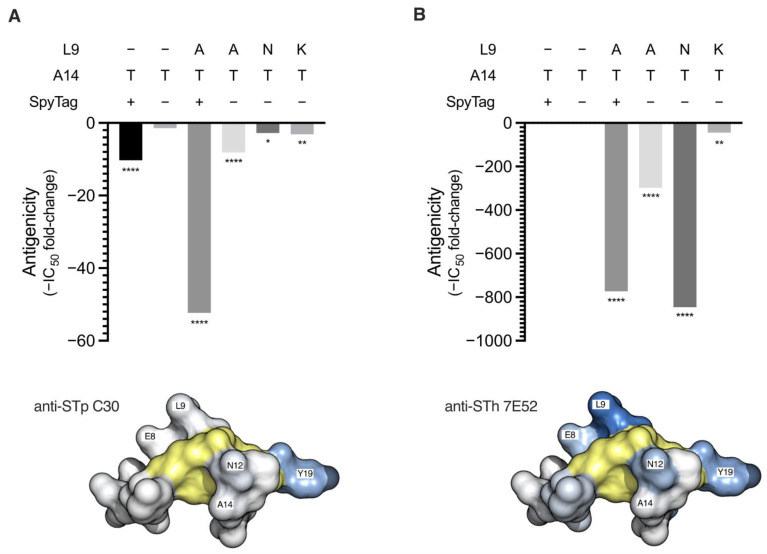
Antigenicity of STh mutant peptides. The antigenicity of the STh mutant peptides towards the anti-STp C30 (**A**) and anti-STh 7E52 (**B**) mAbs were tested using competitive ELISAs. Mutations carried by the STh mutant peptides and the presence of an N-terminal SpyTag are indicated above the plots. The ELISA plates were coated with native STh peptide, and free native STh (control) or the indicated mutant peptides were used to compete for binding to the coating. The 50% inhibitory concentration (IC_50_) for all peptides were estimated using four-parameter logistic regression analyses, and the bar graphs show the IC_50_ fold-changes of all mutant peptides relative to that of native STh. The fold-changes are plotted as negative values to indicate reduced antigenicity. To test whether the IC_50_ for a given mutant peptide was significantly different from that of native STh, ordinary one-way ANOVA and Dunnett’s multiple comparisons test was performed. Significantly different IC_50_s are indicated below the bars: ****, *p* < 0.0001, **, *p* < 0.01, *, *p* < 0.1. Space-filling structural models are shown below the bar graphs to illustrate the location of the named main epitope residues for each mAb (shades of blue) [22,27]. The six cysteines forming the three-disulfide bridges are shown in yellow.

To assess the impact of the introduced mutations on the antigenicity of the peptides, two anti-ST mAbs with distinct epitopes, anti-STp C30 and anti-STh 7E52, were used in competitive ELISAs [22] (Figure 1). When compared to native STh, the dmST peptides had 3- to 52-fold reductions in C30 antigenicity (Figure 1A) and 44- to 846-fold reductions in 7E52 antigenicity (Figure 1B). The much stronger impact on 7E52 antigenicity reflects the fact that the 7E52 epitope is centered around the L9 residue [27], which is mutated in all dmST peptides (Figure 1B). In contrast, the C30 epitope is centered around Y19 [22] and neither A14 nor L9 are part of the epitope (Figure 1B). Hence, a significant reduction in C30 antigenicity may indicate that the introduced mutation(s) lead to structural changes that could ultimately impact immunogenicity. 

In contrast to the dmST peptides, the untagged STh-A14T peptide had C30 and 7E52 antigenicities that were comparable to those of native STh, suggesting that the introduced mutation did not alter the peptide structure. The tagged SpyT-STh-A14T variant, on the other hand, had significantly reduced C30 antigenicity, which may suggest structural alterations or that the SpyTag to some extent interferes with binding to C30. Although both the untagged STh-L9A/A14T and SpyT-STh-L9A/A14T peptides had significantly reduced C30 and 7E52 antigenicities compared to native STh, the reduction was stronger for the tagged peptide. 

The toxicity of the STh mutant peptides was tested using the T84 cell assay. In contrast to native STh, which produced a cGMP response of 318 nM, all mutant peptides produced responses lower than 3 nM cGMP, suggesting that they are non-toxic.

### 3.2. Construction and Characterization of STh Mutant Immunogens 

**Figure 2:**
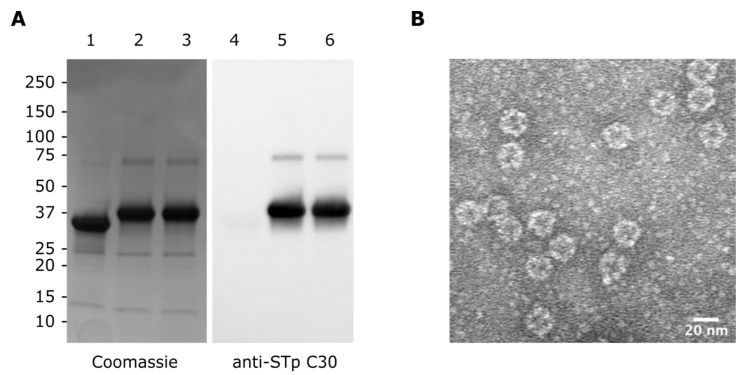
Characterization of STh mutant mi3-nanoparticles. (**A**) Coomassie stained SDS-PAGE (left) and immunoblot (right) of unconjugated mi3-SpyC (lanes 1 and 4) and the mi3-SpyC:SpyT-STh-A14T (lanes 2 and 5) and mi3-SpyC:SpyT-STh-L9A/A14T (lanes 3 and 6) immunogens. Anti-STp mAb C30 was used as primary antibody in the immunoblot. Molecular masses (kDa) of the protein standard are shown to the left. (**B**) Negatively stained transmission electron micrograph of mi3-SpyC:SpyT-STh-L9A/A14T with scale bar (bottom right).

The SpyT-tagged peptides were conjugated to mi3-SpyC by mixing carrier and peptide at 1:3 molar ratios, allowing the spontaneous formation of isopeptide bonds, as documented by SDS-PAGE and immunoblot analysis (Figure 2A). The conjugation reaction was very efficient and resulted in near complete shifts in mobility from 34 kDa of the mi3-SpyC monomer to 38.3 kDa of the mi3-SpyC:SpyT-STh-A14T and mi3-SpyC:SpyT-STh-L9A/A14T conjugate monomers. Since mi3 is a 60-mer nanoparticle, a fully assembled particle displays 60 STh mutant peptide. To verify that mi3 conjugates assembled into nanoparticles, we removed excess peptides and unassembled mi3 monomers by extensive dialysis using a 300 kDa MWCO membrane and subjected the nanoparticles to TEM analysis (Figure 2B). As evident from the micrograph, mi3-SpyC:SpyT-STh-L9A/A14T form uniform particles with a diameter of approximately 25 nm, which is consistent with previous results [20]. 

To make the untagged peptides immunogenic, they were conjugated to BSA using glutaraldehyde. After dialysis to remove unbound peptide, the average carrier:peptide ratios between BSA and mutant peptide were determined by amino acid analysis to be: BSA:STh-A14T, 1:18 (±4.1); BSA:STh-L9A/A14T, 1:17 (±4.3); BSA:STh-L9N/A14T, 1:13 (±3.2); and BSA:STh-L9K/A14T, 1:11 (±4).

### 3.3. Immunization of Mice and Estimation of Anti-STh and Anti-Carrier Titers 

**Figure 3:**
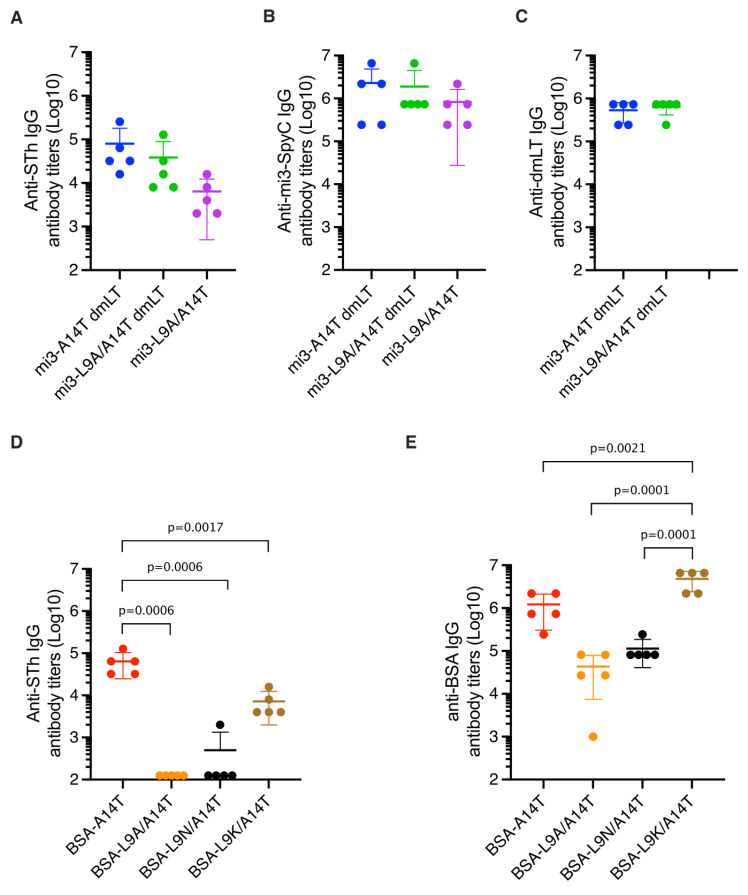
Endpoint IgG antibody titers. The plots show anti-STh IgG (**A**), anti-mi3-SpyC IgG (**B**), and anti-dmLT IgG (**C**) antibody titers of sera from mice immunized with mi3 nanoparticles, and anti-STh IgG (**D**) and anti-BSA IgG (**E**) antibody titers of sera from mice immunized with BSA conjugates. Sera were named according to carrier (BSA or mi3) and STh mutant residues, and dmLT was appended to the name to indicate when dmLT was used as adjuvant. The titers were defined as the highest dilutions that had signal over background ratios of ≥2.1, and the horizontal lines represent the titer mean for each sera group (*n* = 5). Sera means that were statistically significantly different are indicated above the plots by their *p*-values, as estimated using ordinary one-way ANOVA and Tukey’s multiple comparison test. Error bars represent standard deviations (SD) of the means; negative values are not depicted. Additional details of the sera analyses are presented in Table S1.

The mi3-SpyC:SpyT-STh-A14T and mi3-SpyC:SpyT-STh-L9A/A14T nanoparticles were used to immunize five mice each, using dmLT as adjuvant [31]. In addition, five mice were immunized with mi3-SpyC:SpyT-STh-L9A/A14T without any adjuvant. Anti-STh, anti-mi3-SpyC, and anti-dmLT IgG titers were estimated using ELISAs with STh peptide, mi3-SpyC, or dmLT coating, respectively (Figure 3A–C). The mi3-A14T-dmLT sera had the highest mean titers, followed by the mi3-L9A/A14T-dmLT sera. Not unexpected, the unadjuvanted mi3-L9A/A14T sera had the lowest mean titers. The differences in mean anti-STh titers were consistent with the observed differences in anti-mi3-SpyC titers, and the anti-dmLT IgG titers were similar both within and between the adjuvanted groups. 

Each of the BSA conjugates was also used to immunize five mice, and anti-STh and anti-BSA IgG titers were estimated using ELISAs with STh peptide or BSA coating, respectively (Figure 3D,E). The mean anti-STh titers of the BSA-A14T sera were the highest, followed by the BSA-L9K/A14T sera. No anti-STh antibodies were detected in the BSA-L9A/A14T sera (titers < 250), and only one of five BSA-L9N/A14T sera contained detectable anti-STh antibodies. These two groups of sera also had the lowest mean anti-BSA titers, which may suggest that the BSA:STh-L9A/A14T and BSA:STh-L9N/A14T conjugate preparations were less immunogenic than the BSA:STh-A14T and BSA:STh-L9K/A14T conjugates. One interesting feature distinguishes BSA:STh-L9K/A14T from the other dmSTh BSA conjugates, namely that the ε-amino group of the introduced lysine residue of STh-L9K/A14T can be used for conjugation, in addition to the N-terminus. 

### 3.4. Mutant-Specific Antibodies Suggest That dmSTh May Contain Neoepitopes 

When introducing mutations to a small peptide like STh, one risks introducing neoepitopes, which may eclipse native epitopes capable of inducing neutralizing antibodies. To investigate whether any of the STh mutants elicited mutant specific antibodies, we compared the IgG titers of anti-native STh and anti-mutant STh antibodies by coating with native and the cognate mutant STh, respectively (Figure S1). The mi3-A14T-dmLT and BSA-A14T sera contained no or very low levels of mutant-specific antibodies, suggesting that the introduced threonine in the A14 position did not form a neoepitope. In contrast, low to moderate levels of mutant-specific antibodies were observed in several sera from mice immunized with dmSTh immunogens, including mi3-L9A/A14T-dmLT serum 1, 4, and 5, mi3-L9A/A14T serum 3 and 5, BSA-L9N/A14T serum 2, and BSA-L9K/A14T serum 1 and 5. The clearest evidence of the formation of a neoepitope was observed with BSA-L9N/A14T serum 5, which contained high titers of mutant-specific antibodies and no detectable anti-native STh antibodies. In conclusion, these results indicate that when introducing a second mutation in STh, the risk of eliciting irrelevant antibodies increases.

### 3.5. Single and Double Mutant STh Immunogens Elicit Toxin-Neutralizing Antibodies 

**Figure 4:**
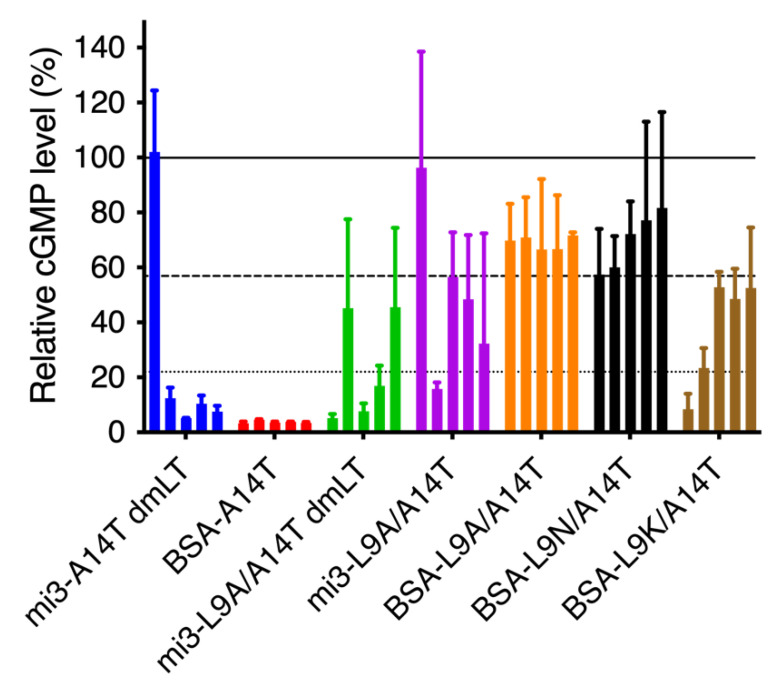
Neutralization of native STh by sera from mice immunized with ST toxoids. Neutralization was assessed using the T84 cell assay, and the estimated cGMP production is given in percent relative to the negative control results where no serum was added (vertical axis). The bars represent the mean results from two separate experiments conducted with technical duplicates, and the error bars indicate the standard deviations. The solid line indicates a relative cGMP level of 100%. The dashed line indicates a relative cGMP level of 57.5%, which was the lowest level observed with a serum containing anti-STh titers < 250 (BSA-L9N/A14T serum 1). Sera with such low levels of antibodies are unlikely to be neutralizing. The dotted line indicates a relative cGMP level of 22.5%, below which the cGMP levels are significantly (*p* < 0.05) different from that of the non-neutralizing BSA-L9N/A14T serum 1, according to a two-way ANOVA followed by Fisher’s LSD test. We consider this to be the threshold below which one can safely conclude that true neutralization is observed.

To assess whether the anti-STh serum antibodies were able to neutralize the toxic activity of native STh, we tested the sera in the T84 cell neutralization assay. Most of the sera appeared to have at least partial neutralizing activity toward native STh (Figure 4), including several sera with anti-STh antibody titers < 250 (Figure 2). The nine sera with anti-STh titers < 250 had relative cGMP levels ranging from 57.5–81.7%, suggesting that in this experiment, cGMP levels above 57.5% should not be considered evidence of neutralizing activity. Hence, only sera with mean cGMP levels significantly (*p* < 0.05) lower than that of the BSA-L9N/A14T serum 1 control (i.e., <22.5%) were considered to have neutralizing activity. This included mi3-A14T-dmLT sera 2–5; all five BSA-A14T sera; mi3-L9A/A14T-dmLT sera 1, 3, and 4; mi3-L9A/A14T serum 2; and BSA-L9K/A14T serum 1. Although not significantly different from the control (*p* = 0.07), one might also consider BSA-L9K/A14T serum 2, with a mean relative cGMP level of 23.5%, to have neutralizing activity. 

### 3.6. Immunological Cross-Reactivity to STp, Uroguanylin, and Guanylin 

**Figure 5:**
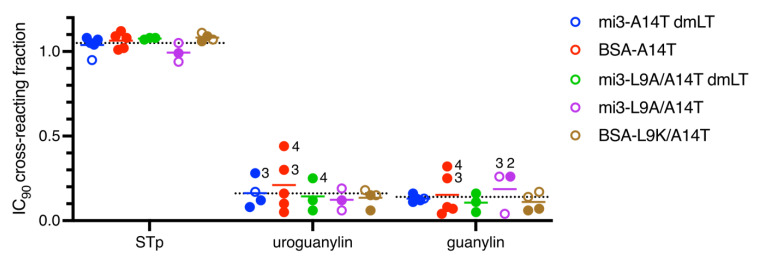
Immunological cross-reactivity of sera towards STp, uroguanylin, and guanylin assessed using competitive ELISAs. For each serum, four-parameter logistic regression was performed, and the 90% inhibitory concentration (IC_90_) was calculated for native STh. These concentrations were then used to calculate the inhibition for each peptide relative to that of native STh. The plots show the cross-reactivity (vertical axis) for each peptide (horizontal axis) in each serum, and the solid lines depict mean cross-reactions within each serum group. For reference, the overall sera mean for each peptide are shown as dotted lines. Sera with neutralizing ability are shown as filled circles, and sera with an IC_90_ cross-reacting fraction >0.2 are indicated by their serum number. Sera with anti-STh titers < 4000 could not be tested for cross-reactivity.

To assess whether the dmSTh immunogens reduced the risk of eliciting unwanted cross-reacting antibodies, we tested whether uroguanylin and guanylin could outcompete binding to native STh in competitive ELISAs (Figure 5). We also included STp in this experiment to assess levels of potentially beneficial cross-reacting antibodies. To ensure consistent comparisons of immunological cross-reactivity between sera, we used the 90% inhibitory concentration (IC_90_) of STh as a common reference point [27], and for each peptide, the cross-reacting fraction of antibodies was calculated by dividing the percent inhibition of the peptide at the reference concentration with that of STh. The overall mean STp-cross-reacting fraction was 1.05 (range 0.99–1.08) (Table S1), with individual sera ranging from 0.94 to 1.12, suggesting strong and consistent cross-reactivity to STp. The overall mean uroguanylin- and guanylin-cross-reacting fractions were 0.16 (range 0.12–0.21) and 0.14 (range 0.11–0.19), respectively, suggesting low levels of unwanted cross-reacting antibodies. However, individual sera with higher levels (>0.2) of cross-reacting antibodies were observed for 1/4 of the mi3-A14T-dmLT sera, 2/5 of BSA-A14T sera, 1/3 of the mi3-L9A/A14T/dmLT sera, and 2/3 of the mi3-L9A/A14T sera. In the mi3-nanoparticle context, individual sera with elevated levels of unwanted cross-reacting antibodies were observed with both single and double mutant STh, and both with and without the dmLT adjuvant. In the BSA-conjugate/Freund’s adjuvant context, the results seem to suggest that the double mutant STh-L9K/A14T had a lower propensity for eliciting unwanted cross-reacting antibodies than the single mutant STh-A14T. However, the BSA-L9K/A14T group only had four sera for which we could assess cross-reactivity, and only two of those neutralized STh. 

Recently, a preclinical characterization of a multivalent ETEC vaccine candidate, MecVax, demonstrated that piglets born to immunized mothers were protected from diarrhea when challenged with an STh-positive ETEC strain [33]. MecVax contains two multivalent proteins: the first is a genetic fusion of adhesin epitopes and the second contains three copies of STh-N12S fused to a monomeric LT-R192G/L211A mutant (3xSTa_N12S_-mnLTR_192G/L211A_). The study did not address whether the 3xSTa_N12S_-mnLTR_192G/L211A_ immunogen elicited antibodies with immunological cross-reactivity towards uroguanylin and guanylin. In an earlier study, 3xSTa_N12S_-mnLTR_192G/L211A_ was compared to similar constructs carrying both double and triple mutant STh variants, and cross-reactivity was assessed using competitive ST ELISA [34]. However, the experimental setup differed from the one used here, as the ratio between free and bound peptide competing for binding to antibody was kept constant. As previously demonstrated [26,27], the measured fraction of antibodies that cross-reacts with uroguanylin or guanylin varies with the competing free peptide dose. Hence, although there appeared to be low levels of cross-reacting antibodies elicited by the genetic fusion constructs, this may be due to the choice of free and bound peptide ratio. In addition, cross-reactivity was assessed on pooled sera, which may mask inter-individual variations.

## 4. Conclusions

This study demonstrates that the SpyCatcher-mi3 nanoparticle platform can be used to make a dmSTh-based vaccine candidate. Neutralizing antibodies were obtained with the double mutant mi3-SpyC:SpyT-STh-L9A/A14T nanoparticles both with and without the dmLT adjuvant. However, the responses varied between individual mice, as reflected by both the anti-STh IgG serum titers and the ability of the sera to neutralize STh. Although dmLT adjuvanted single mutant mi3-SpyC:SpyT-STh-A14T resulted in higher titers and a higher fraction of neutralizing sera than the double mutant nanoparticles, even more consistent responses were obtained in a previous study using unadjuvanted AP205-SpyC:SpyT-STh-A14T VLPs [19]. This suggests that the AP205 VLPs [19], carrying up to 180 STh mutant peptides per particle, may be superior to the mi3 nanovaccine platform [20] that carries 60 STh per particle for constructing an STh-based vaccine component. In addition, AP205 may be more immunogenic by efficiently encapsulating RNA, which can stimulate Toll-like receptor 7 and 8 signaling, and by forming a tightly packed capsid surface in contrast to the porous mi3 nanoparticles [18,20].

The results presented here has strengthened the position of the STh-A14T toxoid as a lead vaccine candidate STh toxoid, since it has elicited neutralizing antibodies when coupled to three different carriers. However, we observed higher levels of unwanted cross-reacting antibodies in this study than in the previous study using non-adjuvanted AP205-STh-A14T [19]. Although choice of carrier seems to have an impact on the risk of eliciting unwanted cross-reacting antibodies, our results also emphasize the need to introduce additional mutations to further reduce that. In that respect, the results on the dmSTh peptides presented here, combining A14T with selected L9 mutations are both encouraging and sobering. The STh-L9K/A14T toxoid seems to elicit lower levels of cross-reacting antibodies than STh-A14T, but the cost seems to be an increased risk for eliciting irrelevant mutant-specific antibodies. We hope that the findings of the present study represent important steps forwards, towards a safe and effective STh-toxoid-based vaccine. 

## Figures and Tables

**Table 1:** List of primers used to generate expression vectors for double mutant STh peptides. Vector sequences are shown in upper case, STh variant coding sequences in lower case, and stop codons are underlined.

Name	Sequence (5’-3’)
L9A/A14T-f	tgtaatcctacatgtaccgggtgctattaaGGCGCCATGGGCAAAGTG
L9A/A14T-r	acatgcttcacagcagtaattgctactattCTGAAAATAAAGATTCTCGCTACCCG
L9N/A14T-f	tgtaatcctacatgtaccgggtgctattaaGGCGCCATGGGCAAAGTG
L9N/A14T-r	acagttttcacagcagtaattgctactattCTGAAAATAAAGATTCTCGCTACCCG
L9K/A14T-f	tgtaatcctacctgtaccgggtgctattaaGGCGCCATGGGCAAAGTG
L9K/A14T-r	acatttttcacagcagtaattgctactattCTGAAAATAAAGATTCTCGCTACCCG
SpyL9A/A14T-f	ctgctgtgaagcgtgttgtaatcc
SpyL9A/A14T-r	taattgctactattACCGG

**Table 2:** Plasmids used to construct and express untagged and tagged STh mutant peptides.

Plasmid	Parent Plasmid	Forward Primer	Reverse Primer	Tag	STh Variant	Ref
pETDsbCin_1b	-	-	-	-	-	[28]
pET-DsbC-STh-A14T	pETDsbCin_1b	-	-	-	STh-A14T	[25]
pET-DsbC-STh-L9A/A14T	pETDsbCin_1b	L9A/A14T-f	L9A/A14T-r	-	STh-L9A/A14T	-
pET-DsbC-STh-L9N/A14T	pETDsbCin_1b	L9N/A14T-f	L9N/A14T-r	-	STh-L9N/A14T	-
pET-DsbC-STh-L9K/A14T	pETDsbCin_1b	L9K/A14T-f	L9K/A14T-r	-	STh-L9K/A14T	-
pET-DsbC-SpyT-A14T	pET-DsbC-STh-A14T	-	-	SpyTag	SpyT-STh-A14T	[19]
pET-DsbC-SpyT-L9A/A14T	pET-DsbC-SpyT-A14T	SpyL9A/A14T-f	SpyL9A/A14T-r	SpyTag	SpyT-STh-L9A/A14T	-

## Supplementary Materials

The following supporting information can be downloaded at: https://www.mdpi.com/article/10.3390/vaccines10020241/s1, Table S1: Serum characteristics. Figure S1: Comparison of anti-mutant STh IgG and anti-native STh antibody titrations. Each row of plots represents the ELISA analyses of sera from five individual mice (numbered 1-5) immunized with one immunogen. Each serum was diluted from 1:250 to 1:256,000 using two-fold dilutions (horizontal axis) and added in parallel to ELISA plates coated with either native STh or cognate STh mutant peptide. The read-out of the ELISA was absorbance at 405 nm (vertical axis), and results are shown by blue circles and lines for anti-mutant STh and red squares and lines for anti-native STh. Sera were named according to carrier (BSA or mi3) and STh mutant residues, and dmLT was appended to the name to indicate when dmLT was used as adjuvant. Error bars depict standard deviations (SD) of triplicate measurements.
