# Supplementary material for: Vaccine Candidate Double Mutant Variants of Enterotoxigenic Escherichia coli Heat-Stable Toxin
Source: Vaccines (Basel). 2022 Feb 4;10(2):241. doi: 10.3390/vaccines10020241 (PMC8874394; doi:10.3390/vaccines10020241)
Supplement: Supplementary file 1 [file vaccines-10-00241-s001.zip › vaccines-1544969-supplementary.pdf]

Table S1. Serum characteristics.

Peptide	Carrier	Adjuvant[a]	Mouse #	Anti-STh titer	Neutralization[b]	IC_{90} cross-reacting fraction[c]	
						Uroguanylin	Guanylin
SpyT-STh-A14T	SpyC-mi3	dmLT	1	16,000	-	0.17	0.13
			2	256,000	12.5 ± 3.8	-	0.16
			3	32,000	5.2 ± 0.1	0.28	0.12
			4	64,000	10.5 ± 2.9	0.12	0.13
			5	32,000	7.6 ± 2.2	0.08	0.11
STh-A14T	BSA	FCA/FIA	1	64000	3.3 ± 0.6	0.1	0.04
			2	128,000	4.5 ± 0.4	0.05	0.07
			3	64,000	3.6 ± 0.4	0.3	0.25
			4	32,000	3.8 ± 0.1	0.44	0.32
			5	32,000	3.4 ± 0.3	0.16	0.08
SpyT-STh-L9A/A14T	SpyC-mi3	dmLT	1	16,000	5.2 ± 1.5	0.06	0.05
			2	8,000	-	-	-
			3	32,000	7.7 ± 2.8	0.12	0.11
			4	128,000	17.0 ± 7.3	0.25	0.16
			5	8,000	-	-	-
SpyT-STh-L9A/A14T	SpyC-mi3	-	1	2,000	-	-	-
			2	8,000	15.9 ± 2.4	0.12	0.26
			3	16,000	-	0.19	0.26
			4	2,000	-	-	-
			5	4,000	-	0.06	0.04
STh-L9A/A14T	BSA	FCA/FIA	1	< 250	-	n.d.	n.d.
			2	< 250	-	n.d.	n.d.
			3	< 250	-	n.d.	n.d.
			4	< 250	-	n.d.	n.d.
			5	< 250	-	n.d.	n.d.
STh-L9N/A14T	BSA	FCA/FIA	1	< 250	-	n.d.	n.d.
			2	2,000	-	n.d.	n.d.
			3	< 250	-	n.d.	n.d.
			4	< 250	-	n.d.	n.d.
			5	< 250	-	n.d.	n.d.
STh-L9K/A14T	BSA	FCA/FIA	1	4,000	8.5 ± 5.6	0.06	0.07
			2	8,000	-	0.15	0.06
			3	16,000	-	0.18	0.14
			4	4,000	-	0.15	0.17
			5	4,000	-	-	-

[a]dmLT, double mutant heat-labile toxin; FCA, Freund's complete adjuvant; FIA, Freund's incomplete adjuvant

[b]Neutralization is reported as percent cGMP level relative to a negative control with no serum added. Only cGMP levels < 22.5 are considered to be neutralizing (see Fig. 5 legend).

[c]90% inhibitory concentration (IC_{90}) of STh was used as a common reference point, and for each peptide, the cross-reacting fraction of antibodies was calculated by dividing the percent inhibition of the peptide at the reference concentration with that of STh.

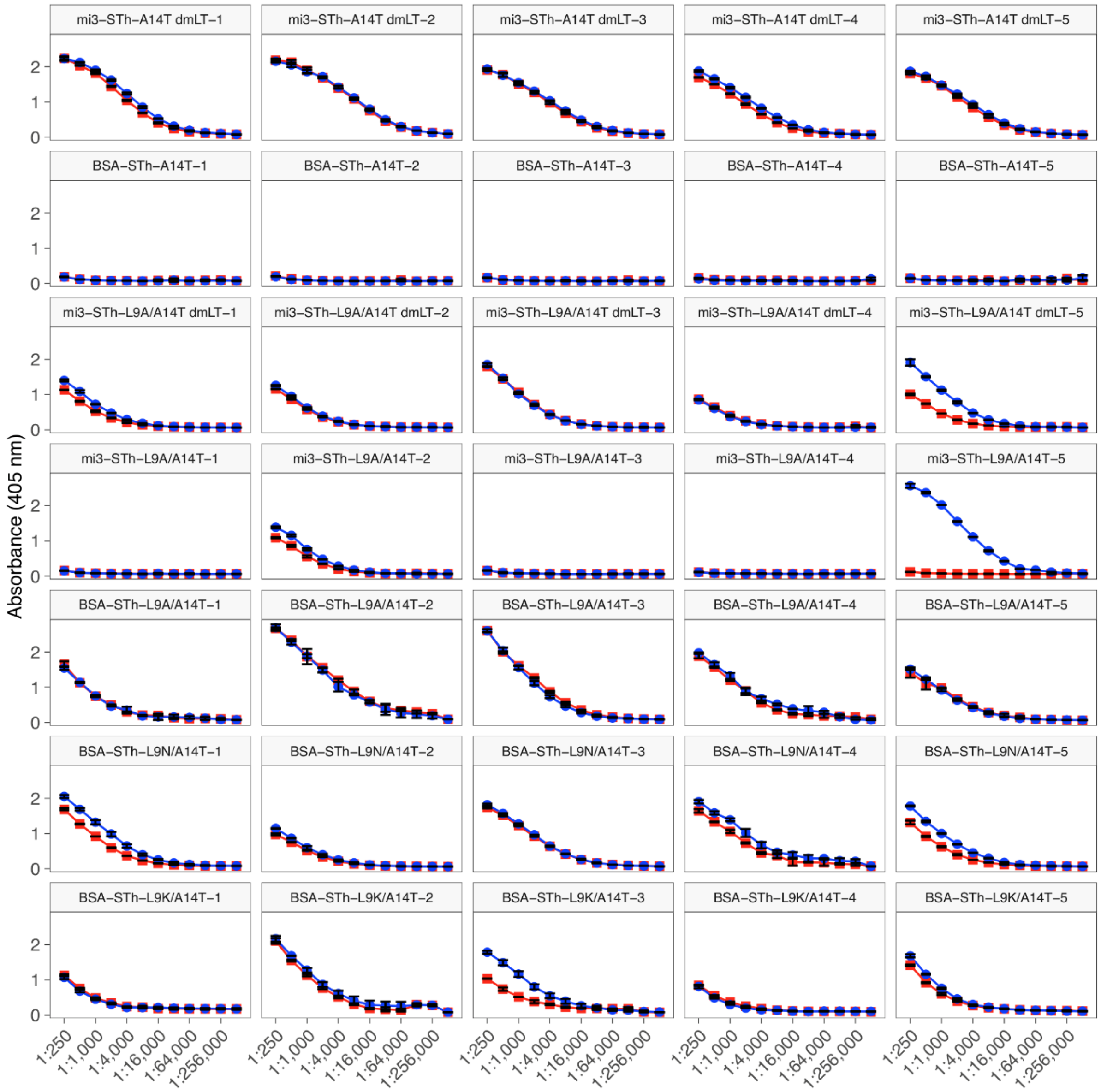

Figure S1. Comparison of anti-mutant STh IgG and anti-native STh antibody titrations. Each row of plots represents the ELISA analyses of sera from five individual mice (numbered 1-5) immunized with one immunogen. Each serum was diluted from 1:250 to 1:256,000 using two-fold dilutions (horizontal axis) and added in parallel to ELISA plates coated with either native STh or cognate STh mutant peptide. The read-out of the ELISA was absorbance at 405 nm (vertical axis), and results are shown by blue circles and lines for anti-mutant STh and red squares and lines for anti-native STh. Sera were named according to carrier (BSA or mi3) and STh mutant residues, and dmLT was appended to the name to indicate when dmLT was used as adjuvant. Error bars depict standard deviations (SD) of triplicate measurements.
